# Iron Fortified Complementary Foods Containing a Mixture of Sodium Iron EDTA with Either Ferrous Fumarate or Ferric Pyrophosphate Reduce Iron Deficiency Anemia in 12- to 36-Month-Old Children in a Malaria Endemic Setting: A Secondary Analysis of a Cluster-Randomized Controlled Trial

**DOI:** 10.3390/nu9070759

**Published:** 2017-07-14

**Authors:** Dominik Glinz, Rita Wegmüller, Mamadou Ouattara, Victorine G. Diakité, Grant J. Aaron, Lorenz Hofer, Michael B. Zimmermann, Lukas G. Adiossan, Jürg Utzinger, Eliézer K. N’Goran, Richard F. Hurrell

**Affiliations:** 1 Laboratory of Human Nutrition, Institute of Food, Nutrition, and Health, ETH Zurich, CH-8092 Zurich, Switzerland; dominik.glinz@usb.ch (D.G.); rita@groundworkhealth.org (R.W.); michael.zimmermann@hest.ethz.ch (M.B.Z.); 2 Basel Institute for Clinical Epidemiology and Biostatistics, University Hospital Basel, CH-4031 Basel, Switzerland; 3 University of Basel, P.O. Box, CH-4003 Basel, Switzerland; juerg.utzinger@swisstph.ch; 4 Université Félix Houphouët-Boigny, 01 BP V34, Abidjan 01, Côte d’Ivoire; mamadou_ouatt@yahoo.fr (M.O.); eliezerngoran@yahoo.fr (E.K.N.); 5 Centre Suisse de Recherches Scientifiques en Côte d’Ivoire, 01 BP 1303, Abidjan 01, Côte d’Ivoire; sebagnoh@gmail.com; 6 Département de Sciologie, Université Alassane Ouattara, 01 BP V18 Bouaké, Côte d’Ivoire; 7 Global Alliance for Improved Nutrition, CH-1202 Geneva, Switzerland; gjaaron@masimo.com; 8 Swiss Tropical and Public Health Institute, P.O. Box, CH-4002 Basel, Switzerland; lhofer92@gmail.com; 9 Hôpital Général de Taabo, Taabo Cité, BP 700 Toumodi, Côte d’Ivoire; adiossanlukas@yahoo.fr

**Keywords:** anemia, cluster-randomized controlled trial, complementary food, Côte d’Ivoire, infant cereal, iron deficiency, iron deficiency anemia, iron fortification, *Plasmodium falciparum*, sodium iron EDTA

## Abstract

Iron deficiency anemia (IDA) is a major public health problem in sub-Saharan Africa. The efficacy of iron fortification against IDA is uncertain in malaria-endemic settings. The objective of this study was to evaluate the efficacy of a complementary food (CF) fortified with sodium iron EDTA (NaFeEDTA) plus either ferrous fumarate (FeFum) or ferric pyrophosphate (FePP) to combat IDA in preschool-age children in a highly malaria endemic region. This is a secondary analysis of a 9-month cluster-randomized controlled trial conducted in south-central Côte d’Ivoire. 378 children aged 12–36 months were randomly assigned to no food intervention (*n* = 125; control group), CF fortified with 2 mg NaFeEDTA plus 3.8 mg FeFum for six days/week (*n* = 126; FeFum group), and CF fortified with 2 mg NaFeEDTA and 3.8 mg FePP for six days/week (*n* = 127; FePP group). The outcome measures were hemoglobin (Hb), plasma ferritin (PF), iron deficiency (ID; PF < 30 μg/L), and anemia (Hb < 11.0 g/dL). Data were analyzed with random-effect models and PF was adjusted for inflammation. The prevalence of *Plasmodium falciparum* infection and inflammation during the study were 44–66%, and 57–76%, respectively. There was a significant time by treatment interaction on IDA (*p* = 0.028) and a borderline significant time by treatment interaction on ID with or without anemia (*p* = 0.068). IDA prevalence sharply decreased in the FeFum (32.8% to 1.2%, *p* < 0.001) and FePP group (23.6% to 3.4%, *p* < 0.001). However, there was no significant time by treatment interaction on Hb or total anemia. These data indicate that, despite the high endemicity of malaria and elevated inflammation biomarkers (C-reactive protein or α-1-acid-glycoprotein), IDA was markedly reduced by provision of iron fortified CF to preschool-age children for 9 months, with no significant differences between a combination of NaFeEDTA with FeFum or NaFeEDTA with FePP. However, there was no overall effect on anemia, suggesting most of the anemia in this setting is not due to ID. This trial is registered at clinicaltrials.gov (NCT01634945).

## 1. Introduction

Iron deficiency (ID) and anemia are considerable public health problems in sub-Saharan Africa [1]. For example, in Côte d’Ivoire, 25–75% of the preschool- and school-age children in rural areas are reported to be iron deficient, and about 80% are anemic [2], which can result in irreversible impairments in cognitive performance and motor development [3,4] unless additional iron is provided. One strategy to provide iron is through iron fortified complementary food (CF). However, this approach is not without challenges due to a large variation in the bioavailability of commonly used iron fortification compounds, frequent unacceptable sensory changes caused by the water soluble iron compounds of highest bioavailability, and the presence of phytic acid (PA), a potent inhibitor of iron absorption in CF containing cereals or legumes [5].

The iron compounds most commonly utilized to fortify CF are ferrous fumarate (FeFum), ferric pyrophosphate (FePP), and electrolytic iron. Ferrous sulfate is less commonly employed as it often causes unacceptable sensory changes in CF [6]. In order to overcome the inhibitory effect of PA on iron absorption, commercially manufactured infant cereals usually contain additional ascorbic acid (AA) at 2:1 molar ratio (AA:Fe), as recommended by the World Health Organization (WHO) [7]. Sodium iron ethylenediaminetetraacetate (NaFeEDTA) is an alternative iron compound that will overcome PA inhibition and is the iron compound of choice for fortifying high PA foods [5]. The Joint Food and Agriculture Organization (FAO)/WHO Expert Committee on Food Additives approved this compound, but proposed an acceptable daily intake of EDTA of 0.2 mg/kg body weight per day which restricts its use in young children [8].

Another potential barrier to the efficacy of iron fortified CF in Côte d’Ivoire is the widespread persistent low-grade inflammation caused by *Plasmodium* spp. (the causative agent of malaria) parasitemia that is reported to decrease iron absorption through increase in hepcidin [9,10]. Previous efficacy studies with iron fortified foods conducted in malaria-endemic settings revealed conflicting results. Iron status improved in young Kenyan children fed NaFeEDTA-fortified maize porridge [11] and in Ivorian school-age children who received meals containing salt fortified with micronized ground FePP [12], which was in line with findings obtained from children in non-malaria endemic areas [13]. In contrast, another trial conducted in Ivorian school-age children found that electrolytic iron fortified biscuits did not improve children’s iron status [14]; the authors suggested this was due to malaria-induced inflammation and use of an iron fortificant with low bioavailability (electrolytic iron) [14].

The aim of this secondary analysis was to determine whether an iron fortified maize-soy CF is efficacious in improving iron status of 12- to 36-month-old Ivorian children in a setting that is hyper-endemic for *Plasmodium falciparum*. We used a locally produced, commercially available fortified CF containing NaFeEDTA and FePP. Additionally, we manufactured a second CF in which FePP was replaced by FeFum. In both CFs, we used the maximum acceptable level of iron as NaFeEDTA (i.e., 2 mg, assuming a mean body weight of 10 kg in our study population of children aged 12–36 months at baseline) [8]. The iron level was completed with 3.8 mg Fe as either FeFum or FePP. Hence, daily feeding of our maize-soy CF provided an extra 5.8 mg iron, equivalent to 100% of the reference nutrient intake (RNI) for one- to three-year-old children assuming an intermediate iron bioavailability of 10% [7]. Children were fed the CF fortified with iron once per day, six days per week, for nine months. Hemoglobin (Hb), iron status (plasma ferritin, PF), prevalence of *P. falciparum*, and inflammation were monitored and compared to a control group of children consuming their normal diet. The main (primary) analysis of the study was to investigate the interaction between iron fortified CF and intermittent preventive treatment (IPT) of malaria in improving Hb concentration. These results have been published elsewhere [15]. In short, no evidence was found for a treatment interaction between IPT and iron fortified CF to increase Hb concentration.

## 2. Subjects and Methods

### 2.1. Study Site and Participants

The study was carried out in the Taabo health and demographic surveillance system (HDSS) in south-central Côte d’Ivoire [16,17]. Located in the transition zone from rainforest (in the South) to savannah (in the North), the Taabo HDSS has ≈43,000 registered inhabitants and is hyper-endemic for malaria [18]. Depending on season and age of the inhabitants, the prevalence of *P. falciparum* ranges from 35–77% [2,19]. Cassava, plantain, and yam are mainly planted for local consumption, whereas coffee and cacao are cash crops. There is a limited amount of fish consumption from local rivers and lakes.

In April 2012, we selected 840 children from five villages (i.e., Ahouaty, Kokoti-Kouamekro, Kotiessou, N’Denou, and Taabo Village) in the designated age range (12–36 months) from the readily available Taabo HDSS database. Children were invited to participate in a baseline screening done from mid-April to mid-May 2012.

### 2.2. Study Design and Procedure

**Figure 1:**
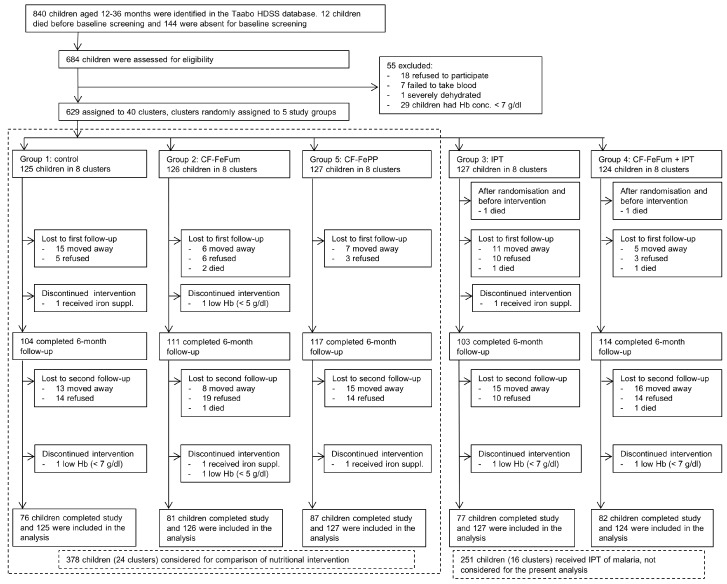
Flowchart. Study groups 1, 2, and 5 were considered for the current secondary analysis. Abbreviations: CF-FeFum, complementary food fortified with NaFeEDTA + ferrous fumarate; CF-FePP, complementary food fortified with NaFeEDTA + ferric pyrophosphate; Hb hemoglobin; HDSS, health and demographic surveillance system; IPT, intermittent preventive treatment of malaria.

We present a secondary analysis of the larger study with key findings published elsewhere [15]. The primary analysis comprised five study groups (Figure 1) and focused on the interaction of iron fortified CF and IPT of malaria on Hb concentration (primary outcome). We selected 629 eligible children at the baseline screening and grouped them into 40 clusters based on proximity of their residence, with at least five clusters in each of the five study villages. Each cluster included between 13 and 18 children. Inclusion criteria were as follows: (i) Hb ≥ 7 g/dL; (ii) no major chronic illnesses, as determined by a study physician; (iii) anticipated residence in the study area for the 9-month intervention; and (iv) no known allergies to albendazole (treatment of choice against soil-transmitted helminthiasis). The clusters were randomly assigned to five study groups by drawing cluster numbers from a hat (urn randomization) together with village authorities. The study groups were: group 1, no nutritional intervention (continuation with the normal diet) and IPT-placebo; group 2, iron fortified CF containing FeFum + NaFeEDTA (FeFum) and IPT-placebo; group 3, no nutritional intervention and IPT of malaria (3-month intervals, using sulfadoxine-pyrimethamine and amodiaquine); group 4, iron fortified CF containing FeFum + NaFeEDTA (FeFum) and IPT of malaria (3-month intervals, using sulfadoxine-pyrimethamine and amodiaquine); and group 5, iron fortified CF containing FePP + NaFeEDTA (FePP) and IPT-placebo. In the current analysis, we focus solely on the iron status of children in the study groups receiving the iron fortified CF (groups 2 and 5) and compare changes in children’s iron status biomarkers over the 9-month intervention with children in the control group (group 1). Hence, 251 children (groups 3 and 4; 16 clusters overall) were excluded from the current analysis.

Study approval was obtained from the institutional review board of ETH Zurich (reference No. EK 2009-N-19) and the national ethics committee of Côte d’Ivoire (reference No. 061 MSLS/CNER). Village authorities, health authorities, and parents/guardians of participating children were informed about the purpose, procedures, and potential risks and benefits of the study. Written informed consent from parents/guardians of the participating children was received. The study progress was assessed by an independent Data and Safety Monitoring Board that provided expertise and evaluated the safety of the study. We registered the trial at clinicaltrials.gov (NCT01634945).

### 2.3. CF Production, Preparation, and Child Feeding

Protein Kissèe-La (Abidjan, Côte d’Ivoire) manufactured the cereal-based, pre-cooked, instant CF. The composition of both study CFs was identical except for the iron compounds. The first study CF-FeFum contained 2.0 mg iron in form of NaFeEDTA and 3.8 mg iron in form of FeFum in a daily serving of 25 g dry weight porridge. The second study CF-FePP is the commercial product from Protein Kissèe-La, sold in Abidjan and contains 2.0 mg iron in form of NaFeEDTA and 3.8 mg iron in form of FePP in the same 25 g serving.

The dried CF contained maize flour (49.9%), soy flour (21.4%), sucrose (20.0%), milk powder (7.0%), aroma (0.3%), salt (0.1%), and a vitamin/mineral premix (1.3%). The content of vitamins and minerals in a 25 g dry weight serving was 667 IU vitamin A, 100 IU vitamin D, 2.5 mg vitamin E, 0.25 mg vitamin B1, 0.25 mg vitamin B2, 0.25 mg vitamin B6, 0.45 μg vitamin B12, 15.0 mg ascorbic acid (AA), 0.075 mg folic acid, 3 mg niacin, 1 mg pantothenic acid, 0.045 mg iodine, 4.15 mg zinc, 0.28 mg copper, 0.6 mg manganese, 66.5 mg calcium, and 0.004 mg biotin. The AA:iron molar ratio was 0.8:1.

Each roller-dried CF was manufactured at three-month intervals during the 9-month study period to avoid losses in product quality during storage. All ingredients were weighed and mixed by Protein Kissèe-La under strict quality control. The manufacturing plant was thoroughly cleaned before CF manufacture. The homogenous mixing of the vitamin/mineral premix into the dry CF powder was assured by quantifying total iron content in three powder samples collected at the onset, in the middle, and toward the end of the mixing. The iron content was measured at ETH Zurich using graphite-furnace atomic absorption spectrophotometry (AA240Z, Agilent Technologies; Santa Clara, CA, USA). The dried CF was filled into 5 kg bags, and each CF was color-coded. The cleaning of the manufacturing plant, mixing, packaging, and labeling was rigorously supervised. After manufacture, Protein Kissèe-La transported the fortified CF to the study site and we stored the dried CFs under temperature-controlled conditions (between 20 °C and max 25 °C). The bags of 5 kg CF were distributed to the villages every other week.

Eight clusters of children (*n* = 125) received no intervention (group 1), eight clusters of children (*n* = 126) received FeFum fortified CF (group 2), and eight clusters of children (*n* = 127) received FePP fortified CF (group 5). These three groups of children received IPT-placebo. One cooking area was installed in each cluster, and women were trained in porridge preparation, including correct dosage (25 mg dry matter), hygiene, and completion of information forms related to food consumption. At each cooking location, the cooking woman prepared the porridge for 13 to 18 children. Children were brought to the cooking areas in the morning and fed by their mothers/guardians under direct supervision. The amount of porridge consumed by each child was recorded daily by the women cooks. Volunteers from the Taabo HDSS monitored cooking locations and cooks daily.

The women cooks used plastic beakers holding approximately 25 g CF powder for dosage. Each porridge serving was individually prepared by mixing 25 g of CF with approximately 100 mL boiled water. Each child consumed the porridge once per day 6 days each week, which provided approximately 34.8 mg added iron per week (5.8 mg added iron per serving). The maize and soy in the CF provided a further 0.6 mg intrinsic iron per serving. The fortificant iron alone provided 100% of the RNI for children aged 1–3 years assuming an intermediate iron bioavailability of 10% [7].

Children assigned to group 1 (control) received no nutritional intervention and continued with their normal dietary habits. Food intake in group 1 children was not monitored, however our previous studies in Côte d’Ivoire reported that young children (2–5 years) in rural areas consume mainly cassava and plantain with sauces based on okra or peanuts [20,21].

### 2.4. Blinding of Treatments

The control group was not blinded to either subjects or investigators. The two CFs were labeled with different colors (red or blue) and were single blinded, whereby the investigator was aware of the product difference. However, the study physician, parents/guardians of children, the cooking women, and the mothers/guardians who administered the porridge were not aware of group assignment.

### 2.5. Follow-Up

Mothers/guardians of participating children were encouraged to refer the child to the nearest health center as soon as the child presented a symptom of illness, especially fever, and to report the sick visit to the Taabo HDSS volunteer in each village. Each child had an individual study identity card. At the time of the study, all health consultations and treatments for children younger than 5 years were free of charge.

### 2.6. Laboratory Methods

Biomedical parameters were monitored at baseline, and after six months and nine months of intervention. We measured Hb, performed a malaria rapid diagnostic test, and prepared a thick and thin blood film from a venous blood sample. α-1-acid-glycoprotein (AGP), C-reactive protein (CRP), and PF were measured in plasma. Details of the laboratory procedures are available elsewhere [15]. We defined anemia as Hb concentration below 11.0 g/dL [22].

CRP above 5 mg/L or AGP concentrations above 1 g/L was considered as inflammation. Due to the high prevalence of inflammation, we defined ID as PF < 30 μg/L [23].

### 2.7. Statistical Analysis

The present report focuses on the impact of iron fortified CF on iron status and includes only three groups (1, 2, and 5) of a 5-arm intervention study. Hb concentration and anemia were our specified primary outcomes. PF concentration, the prevalence of ID, and *P. falciparum* prevalence were secondary outcomes. Results from all five study groups have been previously used to evaluate the interaction of IPT of malaria and iron fortified CF on Hb, anemia, and iron status [15]. The sample size calculation was based on Hb measurements from a 2010 study in preschool-age children in the same region of the Côte d’Ivoire [2]. In this previous study, the mean Hb concentration was 9.7 g/dL with a standard deviation of 2.0 g/dL. We estimated that 125 children per group were needed to detect an Hb difference of 0.8 g/dL at a 90% power level and a 5% level of significance, assuming 20% dropout.

Data were double entered into Microsoft Access 2010 (2010 Microsoft Corporation, Redmond, WA, USA). Double entries were compared using EpiInfo version 3.4.1 (Centers for Disease Control and Prevention, Atlanta, GA, USA) and differences were adjusted according to the original records. Data were analyzed with STATA version 13.1 (StataCorp LP; College Station, TX, USA). For the present analysis, we applied the same statistical approach as for the published paper presenting all five study groups, but restricted the analysis to the three groups receiving the nutritional intervention and corrected for multiple comparison. Briefly, all children randomized into study groups 1, 2, and 5 were analyzed (intention-to-treat). We analyzed the data with mixed (fixed and random) linear regression models to account for random effects due to repeated measures within clusters. The effectiveness was assessed by time-treatment interactions. For the between group comparison at follow-up, time was considered as categorical variable (0, 6, and 9 months). A Bonferroni correction was applied to multiple comparisons. Logistic regression models taking into account random effect were used for analysis of prevalence (i.e., binary) data. Treatment assignment was the fixed effect, and age and CRP concentration were the covariates in all models.

## 3. Results

### 3.1. Participant Characteristics and Compliance

We randomly assigned 378 children (mean age 29.8 (±8.4) months, 50.3% girls) to three study groups. Table 1 shows the biochemical measurements and anthropometry of the children at baseline, and after six months and nine months of intervention. The study period included almost the whole rainy season starting in April and was completed about one month after the onset of the dry season that begins in November. The only difference among the groups at baseline was a significantly higher CRP concentration in the CF-FePP group compared to the control group (*p* = 0.009) and the CF-FeFum group (*p* = 0.013), although the prevalence of inflammation (CRP > 5 mg/L and/or AGP > 1 g/L) did not differ between groups. At baseline, the overall prevalence of anemia, ID, and *P. falciparum* infection among all study children were 82.8%, 34.7%, and 62.0%, respectively. Nearly three-quarters (73%) of the children had elevated inflammation biomarkers (CRP and/or AGP). Helminth infections were rare, with only one child infected with *Ascaris lumbricoides* and another child with *Schistosoma haematobium* at baseline.

The daily amount of uneaten porridge was estimated for each child to the nearest one quarter of a serving. Overall, of the CF-FeFum and CF-FePP groups 92.5% and 94.8%, respectively, of the porridge was consumed.

### 3.2. Hb Concentration and Anemia Prevalence

There were no significant time by treatment interactions on Hb concentrations or anemia (Table 2). There was, however, a significant time effect on Hb concentration and anemia. The increase in Hb of the control group from 9.8 g/dL at baseline to 10.3 g/dL at nine months was not significantly different compared to the increase from 9.9 g/dL to 10.4 g/dL in the CF-FeFum group (*p* = 0.861) and to the increase from 9.6 g/dL to 10.5 g/dL in the CF-FePP group (*p* = 0.430). The change in Hb from baseline to the 9-month follow-up was also not significantly different in both groups receiving iron fortified CF (*p* = 0.535). The decrease of anemia prevalence from baseline to nine months in the CF-FePP group was significantly greater than that in the control group (odds ratio (OR) = 0.42, 95% confidence interval (CI) 0.22–0.83, *p* = 0.036).

### 3.3. PF and Prevalence of ID

There were no differences between PF concentrations in the study groups at baseline. There was no significant time by treatment interaction on PF concentration, but a significant time effect (*p* < 0.001). PF concentrations within groups increased significantly at the two follow-up time points in all study groups. Although the overall time-treatment interaction was not significant, the increase in PF was significantly higher in children from the CF-FeFum group than in the control group at six months (*p* < 0.003), but was not any more significant at nine months of follow-up (*p* = 0.072). The increase of PF concentrations in the FePP group was significantly greater at six months (*p* = 0.048), but was not different from the control at nine months (*p* = 0.150) (Table 2).

There was a significant time by treatment interaction on IDA (*p* = 0.028) and a borderline significant time by treatment interaction on ID (*p* = 0.068) (Table 2). IDA prevalence sharply decreased in the FeFum (32.8% to 1.2%) and FePP groups (23.6% to 3.4%) (for both, *p* < 0.001). The prevalence of ID with or without anemia decreased significantly in the groups receiving the iron fortified CFs; from 40.0% to 3.7% in the group receiving CF-FeFum and from 26.7% to 10.3% in the group receiving CF-FePP (for both, *p* < 0.001). The decrease in ID prevalence observed in the children from CF-FeFum group was significantly greater than the decrease observed in the control group (OR = 0.06, 95% CI 0.02–0.25, *p* < 0.003). Similarly, the decrease in ID prevalence observed in the CF-FePP group was significantly greater than that observed in the control group (OR = 0.17, 95% CI 0.07–0.47, *p* < 0.003). The decreases in ID prevalence observed in the CF-FeFum and CF-FePP were not significantly different from each other (OR = 2.72, 95% CI 0.65–11.41, *p* = 0.171).

### 3.4. P. falciparum Prevalence and Inflammation

The prevalence of *P. falciparum* was around 60% in children from all groups at baseline and the 6-month follow-up (Table 1). *P. falciparum* prevalencedecreased slightly to around 45% at study completion during the dry season. Although there were some small differences in CRP and AGP values in children from different groups at different time points (Table 1), the prevalence of inflammation (CRP > 5 mg/L and/or AGP > 1 g/L) also remained high, ranging between 57.3% and 76.4% (*p* > 0.05), over the 9-month intervention period in children in all groups.

## 4. Discussion

The main finding of this study is that, despite the high *P. falciparum* prevalence and elevated inflammation biomarkers in 12- to 36-month-old children, consumption of iron fortified CF for nine months significantly decreased the prevalence of IDA. Our results indicate that, despite previous reports of decreased iron absorption from labelled single meal studies in adults and children infected with *P. falciparum* [9,24], iron absorption from the iron fortified CF in this study was sufficient to improve iron status and decrease IDA. Our findings suggest that, in the long term, the drive to increase iron absorption during ID can overcome the restriction in iron absorption from inflammation. This observation is consistent with previous studies showing that iron absorption is up-regulated in children with low iron status regardless of their inflammation status [25,26]. In addition, in a recent study in Gambian and Tanzanian children, hepcidin concentration was lower in iron deficient children infected with *P. falciparum* than in children of normal iron status infected with *P. falciparum* [27].

Although there was a significant reduction in IDA in this study, there was no significant overall reduction in anemia. This suggests that the major cause of anemia in this age group in this setting is not iron deficiency (ID). We have previously reported that anemia prevalence was high in our preschool-age children in rural Côte d’Ivoire and it was strongly linked to *P. falciparum* infection [2]. In our study, anemia prevalence was around 80% at baseline. Approximately 60% of our children were infected with *P. falciparum* at baseline and parasitemia remained approximately at the same level throughout the first six months during the rainy season. *Plasmodium falciparum* prevalence slightly declined to less than 50% at the end of the study in the dry season (Table 1). Between 57% and 77% of the children presented with elevated CRP and/or AGP at baseline, after six months, and after nine months. As inflammation, via its action on increasing hepcidin, would be expected to inhibit the recycling of red cell iron [28], much of the anemia in our study children would be expected to be anemia of inflammation, likely due to an infection with *P. falciparum* or other infectious agents. The lack of a substantial rise in overall Hb concentrations over the nine months of the study, despite an increase in iron stores, indicates that iron availability to the bone marrow for erythropoiesis was likely limited in many children by high rates of infection leading to hepcidin-mediated iron sequestration in macrophages of the reticulo-endothelial system. In addition, suppression of erythropoiesis and shortening of the red blood cell life span by malaria and infectious inflammation likely contributes to anemia in these children. High circulating hepcidin concentrations also decrease dietary iron absorption by inactivating the iron export protein ferroportin in duodenal enterocytes [29]. Previous absorption studies in women and children with *P. falciparum* parasitemia showed that iron absorption is only about half of the absorption measured after receiving antimalarial treatment [9,10]. Moreover, the malaria pigment hemozoin, a side product of the Hb digestion by *P. falciparum,* directly inhibits the erythropoiesis in the bone marrow [30]. It remains unclear whether an increase in iron stores in children in malaria-endemic settings, without an increase in Hb, will lead to an improvement in health. It is likely that a portion of this extra iron would be utilized by the iron requiring enzymes of the brain, energy metabolism, and immune system. Nevertheless, it appears that the children in our study were still able to absorb some iron from the iron fortified CF, despite the inhibitory effects of malaria and infectious inflammation.

At baseline, 24–33% of the children had ID in the presence of anemia. While ID could have been an additional cause of the observed anemia, it is conceivable that a considerable amount of the observed anemia is due to inflammation either separately or concurrent with IDA. Inherited hemoglobinopathies are unlikely to contribute to anemia, as a previous study in the Taabo HDSS reported 83% of subjects to have normal Hb genotype, and only 7%, 8%, and 1% carried the C allele, S allele, or had sickle cell anemia, respectively [2]. Vitamin A deficiency has been reported to be prevalent, but was not associated with anemia in young children [2] and infections with soil-transmitted helminths or *Schistosoma* spp. were rare in our study cohort, and thus unlikely to contribute to anemia in a substantial manner.

The inability of iron fortified foods to impact on anemia in malaria-endemic settings may be partly related to the relatively low amount of iron provided. The higher levels of iron used in iron supplements [31] and added to micronutrient powders [32] used for home fortification have been reported to decrease anemia prevalence in children in malaria-endemic areas. The lower levels of iron in fortified foods have two advantages. First, unlike with iron supplementation, there is no spike in iron absorption so there is little or no formation in plasma of non-transferrin bound iron, which is considered responsible for increasing the severity of *P. falciparum* infection [33,34]. Second, less unabsorbed iron reaches the colon, so there is a lower risk of changing the gut microbiome to a more pathogenic profile [35].

The fortified CF provided each child with its RNI for iron (5.8 mg added iron) for six days each week. Based mainly on adult absorption studies, the relative iron absorption from NaFeEDTA added to cereal foods would be expected to be 2–3 times greater than that from FeFum and 4–6 times greater than that from FePP [5]. It was somewhat surprising therefore that the CF fortified with FePP was as efficacious in improving iron status as the CF fortified with FeFum. One likely explanation is that the fortified CF provided considerably more iron than the gap between the child’s iron intake from their regular diet and the child’s daily iron requirement. Earlier studies from our group in Côte d’Ivoire reported that preschool-age children (2–5 years) consumed 5.5 mg Fe per day [20] which is close to the RNI of 5.8 mg recommended for one- to three-year-old children and to the 6.3 mg iron recommended for four- to six-year-old children assuming 10% iron absorption [7]. The iron bioavailability of the diet is not known but could approach 10% as the main staples of cassava and plantain are low in PA. It is possible therefore that the 2 mg of highly bioavailable iron from NaFeEDTA provided most or all of the iron lacking in the diet, and that any difference in iron absorption from CF-FeFum and CF-FePP had little or no effect. In Côte d’Ivoire, the commercial CF is identical to the CF (FePP) used in the present study. Our study confirms that this formulation will benefit the child’s iron status. 

Our study has several strengths. It was conducted in infants and young children in a rural region of West Africa where anemia and ID are very common and where *P. falciparum* is hyperendemic. We used iron compounds with highly bioavailable NaFeEDTA, and we maximized its content and completed with one of the two compounds, FeFum or FePP, which have relatively good bioavailability. It remains unclear whether most of the iron was absorbed from NaFeEDTA and whether by only using NaFeEDTA the effect would have been the same; this should be addressed in future research. Limitations of our study include possible confounding by seasonal variations in malaria transmission, the use of a single iron biomarker (PF) to define iron status, and the lack of plasma hepcidin measurements. A further limitation is that we did not consistently record potential adverse events in the different intervention groups because of limited financial and human resources and at times difficult access to some of the most remote communities. 

In conclusion, our study confirms the high prevalence of anemia (> 80%) and ID (> 30%) in young children living in rural Côte d’Ivoire. This is a major public health concern that needs to be urgently addressed. Iron fortified, cereal-based CFs are highly efficacious in correcting IDA in young children living in malaria-endemic settings. Our study shows the usefulness of using the maximum amount of NaFeEDTA as permitted by its acceptable daily intake and completing the iron fortification level with other iron compounds. The current study, however, found that iron-fortified foods had no significant overall impact on anemia, presumably because the anemia is mainly attributable to malaria and infectious inflammation, and not ID. Our findings highlight the importance of assessing the etiology of anemia in order to design and implement appropriate intervention(s) [36]. Further research is needed to better understand the overlapping causes of anemia, and to develop methods to quantify ID in the presence of malaria-induced inflammation.

## Figures and Tables

**Table 1:** Between group comparison of anthropometric measures, *P. falciparum* infection prevalence, *P. falciparum* parasitemia, and inflammation biomarkers and prevalence at baseline, six months, and nine months in 12- to 36-month-old Ivorian children fed iron fortified complementary food (CF) containing NaFeEDTA combined with either ferrous fumarate (FeFum) or ferric pyrophosphate (FePP).

Groups			
	Control	CF-FeFum	CF-FePP
	**Participants N =**		
Baseline	125	126	127
6 months	104	111	116
9 months	76	81	87
	**Height** (cm, mean, SD)		
Baseline	79.2 ± 9.8	78.5 ± 7.5	78.6 ± 6.8
6 months	86.2 ± 6.9	86.8 ± 6.9	85.8 ± 6.6
9 months	89.0 ± 6.6	89.3 ± 6.5	89.0 ± 6.3
	**Body weight** (kg, mean, SD)		
Baseline	10.7 ± 2.3	10.8 ± 2.9	10.7 ± 2.5
6 months	11.2 ± 2.0	11.1 ± 2.0	11.0 ± 1.8
9 months	11.7 ± 2.1	11.5 ± 1.9	11.4 ± 1.7
	***P. falciparum* prevalence**		
Baseline	62.1%	57.7%	66.1%
6 months	62.5%	55.0%	64.7%
9 months	44.7%	46.9%	47.1%
	***P. falciparum* parasitemia** (parasites/μL blood, geometric mean, 95% confidence interval)		
Baseline	1136 (729–1768)	896 (524–1534)	2182 (1409–3379)
6 months	3773 (2470–5762)	2268 (1427–3605)	2074 (1367–3146)
9 months	2820 (1460–5447)	2718 (1662–4445)	3130 (1913–5121)
	**CRP** (mg/L, median, interquartile range 25th 75th)		
Baseline	2.8 (1.0–11.1)	3.4 (1.4–8.7)	5.9 (1.9–21.3) *^ᴪ^
6 months	5.1 (1.8–18.6)	4.6 (1.2–20.4)	4.8 (1.5–14.6) **
9 months	2.6 (1.0–7.3)	4.3 (1.0–13.2)	3.2 (1.3–14.8) ^ᴪ^
	**AGP** (g/L, median, 25th 75th)		
Baseline	1.12 (0.90–1.40)	1.27 (1.01–1.54)	1.26 (0.96–1.65)
6 months	1.13 (0.88–1.41)	1.25 (0.92–1.54)	1.10 (0.85–1.40)
9 months	1.07 (0.78–1.44)	1.13 (0.85–1.36)	1.06 (0.79–1.28) *
	**Prevalence of inflammation** (CRP > 5 mg/L and/or AGP > 1 g/L)		
Baseline	65.8%	76.8%	76.4%
6 months	72.1%	74.8%	65.5%
9 months	57.3%	64.2%	57.5%

Changes between baseline to six months and baseline to nine months were compared between groups with random effect models. Abbreviations: AGP, α-1-acid-glycoprotein; CRP, C-reactive protein; Hb, hemoglobin; SD, standard deviation. * *p* < 0.05 and ** *p* < 0.01 significant difference at baseline or increase/decrease in CF-FeFum or CF-FePP significantly different compared to increase/decrease in control. ^ᴪ^ *p* < 0.05 significant difference at baseline or increase/decrease in CF-FePP significantly different compared to CF-FeFum.

**Table 2:** Hemoglobin (Hb) concentration and iron status biomarkers at baseline, six months, and nine months in 12- to 36-month-old Ivorian children in the control group and in children consuming iron fortified complementary food (CF) containing NaFeEDTA combined with either ferrous fumarate (FeFum) or ferric pyrophosphate (FePP).

	Groups			Overall Effects			Between Group Comparisons		
	Control	CF-FeFum	CF-FePP	Time *p* =	Treatment *p* =	Time by Treatment Interaction *p* =	Control vs. CF-FeFum *p* =	Control vs. CF-FePP *p* =	CF-FeFum vs. CF-FePP *p* =
	**Participants N =**								
Baseline	125	126	127						
6 months	104	111	116						
9 months	76	81	87						
	**Hb concentration** (g/dL, mean, ±SD)								
Baseline	9.8 ± 1.3	9.9 ± 1.2	9.6 ± 1.2	**<0.001**	0.948	0.141			
6 months	9.9 ± 1.3	9.9 ± 1.3	10.0 ± 1.1				0.761	0.479	0.306
9 months	10.3 ± 1.3 *	10.4 ± 1.2 *	10.5 ± 1.2 **				0.871	0.226	0.161
	**Anemia** (Hb < 11 g/dL)								
Baseline	81.6%	80.2%	86.6%	**<0.001**	0.475	0.237			
6 months	79.8%	77.5%	81.9%				0.216	0.953	0.746
9 months	71.1%	70.4%	65.5%*				0.083	**0.036 ^a^**	0.069 **^a^**
	**PF** (μg/L, median, interquartile range 25th 75th)								
Baseline	37.7 (18.3–72.4)	36.2 (21.6–66.0)	53.0 (28.4–115.7)	**<0.001**	0.068	0.458			
6 months	60.7 (35.1–114.0) ***	102.4 (48.3–159.5) ***	69.1 (41.8–139.7) ***				**<0.003 ^a^**	**0.048 ^a^**	0.214
9 months	49.6 (26.2–96.0) **	66.5 (45.4–117.4) ***	62.6 (41.1–107.2) ***				0.072 **^a^**	0.150	0.426
	**ID** (PF < 30 µg/L)								
Baseline	37.4%	40.0%	26.7%	**<0.001**	**0.004**	0.068			
6 months	19.2% *	5.4% ***	8.6% ***				**<0.003 ^a^**	**0.006 ^a^**	0.368
9 months	29.3%	3.7% ***	10.3% ***				**<0.003 ^a^**	**0.003 ^a^**	0.171
	**IDA** (PF < 30 μg/L and Hb < 11 g/dL)								
Baseline	33.3%	32.8%	23.6%	**<0.001**	**0.003**	**0.028**			
6 months	15.4% **	3.6% ***	4.3% ***				**0.036 ^a^**	0.096 **^a^**	0.689
9 months	18.7%	1.2% ***	3.4% ***				**0.027 ^a^**	**0.018 ^a^**	0.388
	**ID and inflammation** (PF < 30 μg/L and inflammation: CRP > 5 mg/L and/or AGP > 1 g/L)								
Baseline	21.1%	24.0%	14.2%	**<0.001**	**0.001**	0.080			
6 months	11.5% *	4.5% ***	4.3% ***				0.099 ^a^	**0.096 ^a^**	0.900
9 months	14.7%	2.5% **	2.3% **				**0.033 ^a^**	**0.021 ^a^**	0.919
	**Anemia, ID, and inflammation** (Hb < 11 g/dL and PF < 30 µg/L and inflammation: CRP > 5 mg/L and/or AGP > 1 g/L)								
Baseline	18.7%	20.8%	13.4%	**<0.001**	**0.003**	**0.040**			
6 months	10.6%	2.7% **	3.4% ***				**0.048 ^a^**	0.090 ^a^	0.513
9 months	12.0%	1.2% **	1.1% **				0.063 ^a^	**0.048 ^a^**	0.944

^a^ Changes between baseline to six months and baseline to nine months were compared between groups with random effect models. For the between group comparison at follow-up, time (0, 6, and 9 months) was considered as categorical variable, For group differences with significant *p*-values (<0.05) a Bonferroni correction was applied. Abbreviations: AGP, α-1-acid-glycoprotein; CRP, C-reactive protein; Hb, hemoglobin; PF, plasma ferritin; SD, standard deviation. * *p* < 0.05, ** *p* < 0.01, *** *p* < 0.001 significant difference within the same group from baseline to six months or baseline to nine months.

## Abbreviations

AA	ascorbic acid
AGP	α-1-acid glycoprotein
CF	complementary food
CRP	C-reactive protein
FAO	Food and Agriculture Organization
Fe	iron
FeFum	ferrous fumarate
FePP	ferric pyrophosphate
Hb	hemoglobin
HDSS	health and demographic surveillance system
ID	iron deficiency
IDA	iron deficiency anemia
IPT	intermittent preventive treatment
NaFeEDTA	sodium iron ethylenediaminetetraacetate
PA	phytic acid
PF	plasma ferritin
RNI	reference nutrient intake
WHO	World Health Organization
